# Introduction of an Extracorporeal Cardiopulmonary Resuscitation Eligibility Protocol for Paramedics in Atlantic Canada: A Pilot Knowledge Translation Project

**DOI:** 10.7759/cureus.6185

**Published:** 2019-11-18

**Authors:** Colin P Rouse, Jay Mekwan, Paul Atkinson, Derek Rollo, Jacqueline Fraser, Joanna Middleton, Tushar Pishe, Michael Howlett, Sohrab Lutchmedial, Jean-François Légaré, Steve Chanyi, Mark Tutschka, Ansar Hassan, James Gould

**Affiliations:** 1 Emergency Medicine, Queen Elizabeth II Health Sciences Centre, Halifax, CAN; 2 Emergency Medicine, Horizon Health Network, Saint John, CAN; 3 Emergency Medicine, Saint John Regional Hospital, Saint John, CAN; 4 Family Medicine, Saint John Regional Hospital, Saint John, CAN; 5 Emergency Medicine, Saint John Regional Hospital / Horizon Health Network, Saint John, CAN; 6 Interventional Cardiology, Saint John Regional Hospital / Dalhousie University, Saint John, CAN; 7 Cardiac Surgery, Saint John Regional Hospital / Dalhousie University, Saint John, CAN; 8 Cardiac/Thoracic/Vascular Surgery, Saint John Regional Hospital, Saint John, CAN; 9 Critical Care Medicine, Saint John Regional Hospital / Dalhousie University, Saint John, CAN; 10 Emergency Medicine, Queen Elizabeth II Health Science Center / Dalhousie University, Halifax, CAN

**Keywords:** cardiac arrest, ecpr, prehospital, paramedic, simulation, ecmo

## Abstract

Introduction

There is currently no protocol for the initiation of extracorporeal cardiopulmonary resuscitation (ECPR) for out of hospital cardiac arrest (OHCA) in Atlantic Canada. Advanced care paramedics (ACPs) perform advanced cardiac life support in the prehospital setting often completing the entire resuscitation on-scene. Implementation of ECPR will present a novel intervention that is only available at the receiving hospital. Our objective is to determine if an educational program can improve identification of ECPR candidates by paramedics. Establishing paramedic competence will ensure rapid transfer of eligible patients for a potentially life-saving intervention.

Methods

An educational program was delivered to paramedics including a short seminar and pocket card coupled with simulated OHCA cases. A before-and-after study design using a case-based survey was employed. Paramedics were scored on their ability to correctly identify patients suffering OHCA who met the inclusion criteria for our ECPR protocol. A Wilcoxon matched-pairs signed rank test was employed to compare paramedics’ scores before and after the education delivery. A six-month follow-up is planned to assess retention. Qualitative data was also collected from paramedics during simulation to help identify practical issues, potential barriers, and to refine inclusion and exclusion criteria prior to the implementation of our protocol in the prehospital setting.

Results

The median score pre-education was 10 (IQR: 9-10.5) compared to 14 (IQR: 13-15) after education delivery. The median difference between groups was 5. The Wilcoxon matched-pairs test demonstrated a significant improvement in the paramedics’ ability to correctly identify ECPR candidates after completing our educational program z = -2.67, p = 0.0039.

Conclusion

Paramedic training through a didactic session coupled with a pocket card and simulation appeared to be a feasible method of knowledge translation. Six-month follow-up data will help ensure knowledge retention is achieved.

## Introduction

In recent years there has been a paradigm shift towards advanced training for paramedics. Providers such as advanced care paramedics (ACP) and critical care paramedics (CCP) have acquired advanced skills including endotracheal intubation, administration of fluids, and delivery of various medications. These advanced skills have been shown to be advantageous in certain patient populations. For example, a study in Scotland demonstrated that prehospital thrombolysis delivered by paramedics shortened time to thrombolytic in patients with out of hospital ST-elevation myocardial infarction [1].

Alternatively, in out of hospital cardiac arrest (OHCA), it has been suggested that basic life support (BLS) with a focus on early defibrillation may improve outcomes [2]. Although several advanced prehospital treatments and interventions have been studied, they fail to reliably improve outcomes [3]. Traditionally in our region ACPs spend more time on scene than primary care paramedics (PCP), as they can deliver advanced interventions. A large multi-centre trial examined the effect of scene time on neurological outcome in patients surviving OHCA. Their findings suggest that an intermediate scene time of 4-7 mins results in the best neurologic outcomes in patients who could not be resuscitated in the field [4]. It is known that survival of OHCA improves with shorter emergency medical services (EMS) response times, EMS cardiopulmonary resuscitation (CPR), and early defibrillation [5]. Based on these findings it has been postulated that optimal treatment of patients suffering OCHA should continue to emphasize early response times, quality CPR and early defibrillation. What has remained less clear is the optimal care strategy for patients in whom these early interventions fail.

Emerging evidence suggests that extracorporeal cardiopulmonary resuscitation (ECPR) applied to specific patient populations in a timely manner can improve both survival, and survival with a good neurological outcome in OHCA [6-8]. Currently, at our institution and in our prehospital EMS system there is no protocol for initiation of ECPR for refractory OHCA. Our goal was to develop a feasible knowledge translation tool for paramedic triage of potential ECPR patients. This pilot project was limited to advanced care paramedics to allow for increased feasibility with the smaller sample of available ACPs in our region. If successful, the educational tool will be implemented to include all prehospital providers. This is of great importance in our prehospital system as there currently is not 24-hour coverage of ACPs in the community and they are not always available to respond to the OHCA calls. Should we wish to provide 24-hour ECPR coverage, all prehospital providers will have to be trained to appropriately identify ECPR candidates. The current study addressed the first phase of this potential protocol, patient selection, and attempted to identify if an educational package consisting of a didactic teaching session, an educational pocket card, and simulated OHCA cases can improve paramedic ability to correctly identify ECPR candidates.

## Materials and methods

Study Design

This study used a standard before-and-after design which examined the performance of ACPs in appropriately identifying patients who met inclusion criteria for ECPR delivery as well as correctly excluding patients based on exclusion criteria. Performance was tested before and after the delivery of an educational training session. This educational program included a pocket card, and a didactic teaching session followed by three high fidelity simulations. A survey composed of clinical vignettes was performed using Google Forms to test the paramedics' ability to correctly triage patients (Appendix).

Setting

Saint John is a coastal city located in the Atlantic Canadian province of New Brunswick. It is the second largest metropolitan area in New Brunswick with a population of 126,202 persons [9]. The Saint John Regional Hospital is a community and teaching hospital with approximately 445 beds and 48,000 visits to the emergency department each year [10,11]. It is the only level 1 trauma centre in New Brunswick and is also the location of the New Brunswick Heart Centre, the only percutaneous coronary intervention (PCI) capable hospital in New Brunswick.

New Brunswick has a provincial EMS comprised of mostly PCPs, however ACPs have recently been introduced into the provincial prehospital care system on a trial basis. In cases of suspected OHCA the closest available paramedic team is dispatched to the scene. If the initial ambulance on scene consists of PCPs they will treat the patient according to their BLS treatment algorithm and an additional ACP unit will be dispatched when available. The ACP unit then either travels to the scene of the ongoing resuscitation or meets the PCP ambulance during transport to hospital. After arrival to the patient the ACP team then assumes the lead for the resuscitation according to their advanced life support (ALS) treatment pathway. Each treatment pathway has its own criteria for patient transport and termination of resuscitation. Data in the provincial ambulance database demonstrated a trend of increased scene time in OHCA when the treating paramedics were ACPs.

The ACP treatment protocol for OHCA was developed based on the assumption that ALS providers can perform initial resuscitation that is equivalent to an in-hospital resuscitation attempt. Therefore, longer scene times have been generally accepted. While this assumption was likely true at the time of its development, the introduction of an ECPR protocol makes available a new time sensitive in-hospital intervention. This novel intervention, extracorporeal membrane oxygenation (ECMO), could improve outcomes in certain patients suffering refractory OHCA.

Data Collection and Analysis

Ethical approval was sought prior to the collection of any data. Ethical approval was granted by the Horizon Health Research Ethics Board (REB File #: 2017-2487). ACPs were recruited on a voluntary basis to complete a pre-survey by email or in person prior to participation in training. A didactic teaching session on ECPR and the eligibility criteria for our centre were provided. A description of the study and a declaration of consent were provided on the initial page of the survey before any survey content was displayed. No personally identifiable data was collected. Participants were asked to create a unique identifier to avoid duplication of data. Two surveys were distributed to the ACPs. The pre-survey presented several clinical cases with patients randomly organized as either eligible or non-eligible for ECPR. The eligibility criteria can be viewed in Table 1. The same survey presented in a random order was then distributed after the paramedics completed their education. A Wilcoxon matched-pairs signed rank test was performed to compare the pre-education survey results to the post-education results to determine if the paramedics improved their ability to correctly identify patients eligible for ECPR. Undertriage and overtriage rates were calculated according to the formula proposed by Peng and Xiang [12].

**Table 1:** Eligibility criteria. CPR: Cardiopulmonary resuscitation; ROSC: Return of spontaneous circulation; PEA: Pulseless electrical activity; VT: Ventricular tachycardia; VF: Ventricular fibrillation.

Inclusion Criteria	Exclusion Criteria
1. Witnessed out of hospital cardiac arrest	1. Unwitnessed cardiac arrest
2. Age 18-70	2. Asystole after initial resuscitation (at the time of transport decision)
3. No flow time < 10 mins (from arrest to initiation of CPR)	3. Suspected Etiology:
4. 10 minutes or 3 rounds of CPR completed (whichever comes first) without ROSC	A) Uncontrolled bleeding
5. PEA/VT/VF as heart rhythm	B) Irreversible brain damage
6. Mechanical chest compression device available	C) Trauma
7. Cardiac catheterization Lab open (7 am – 7 pm)	4. Comorbidities:
8. ≤20-minute transport time to hospital	A) Standing do-not-resuscitate order
	B) Undergoing end-of-life care
	C) Unable to fit mechanical chest compression device on patient
	5. Pregnancy

## Results

A total of nine participants were included in this pilot project. Eight participants had the designation of advanced care paramedic and one participant held the designation of critical care paramedic. A summary of demographic information can be found in Table 2.

**Table 2:** Demographics ACP: Advanced care paramedic; CCP: Critical care paramedic.

Demographics			
	Male, n (proportion)	Female	
Gender	7 (0.78)	2 (0.22)	
	25-34	35-44	45-54
Age	2 (0.22)	6 (0.67)	1 (0.11)
	ACP	CCP	
Level of Training	8 (0.89)	1 (0.11)	

**Figure 1:**
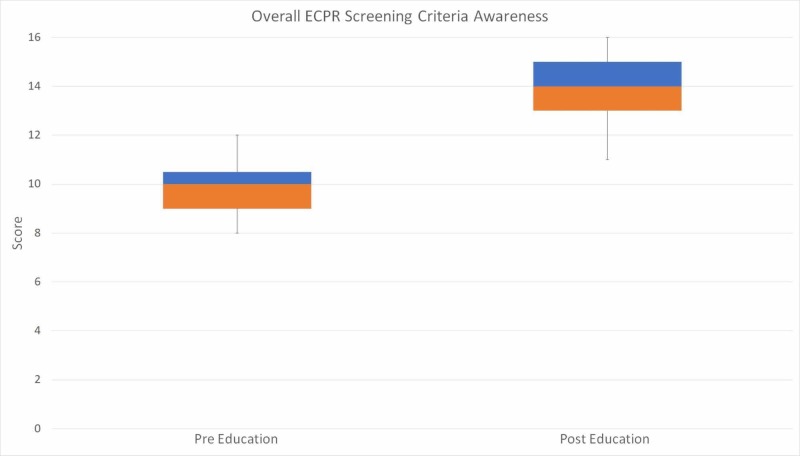
Whisker plot representation of the median, IQR, and range of paramedic performance both pre- and post-education delivery. ECPR: Extracorporeal cardiopulmonary resuscitation; IQR: Inter-quartile range.

Paramedics scored a median of 10 (IQR: 9-10.5) in the pre-education survey. This was compared to the median score of 14 (IQR: 13-15) in the post-education survey. The median difference between the pre- and post-education survey was five. The primary outcome of interest, correct triage of ECPR eligible patients was significantly improved after the education delivery as demonstrated by the results of the Wilcoxon matched-pairs signed rank test, z = -2.67, p = 0.0039. A graphical representation of the paramedics’ performance through their quartiles is displayed in the whisker plot presented in Figure 1.

**Figure 2:**
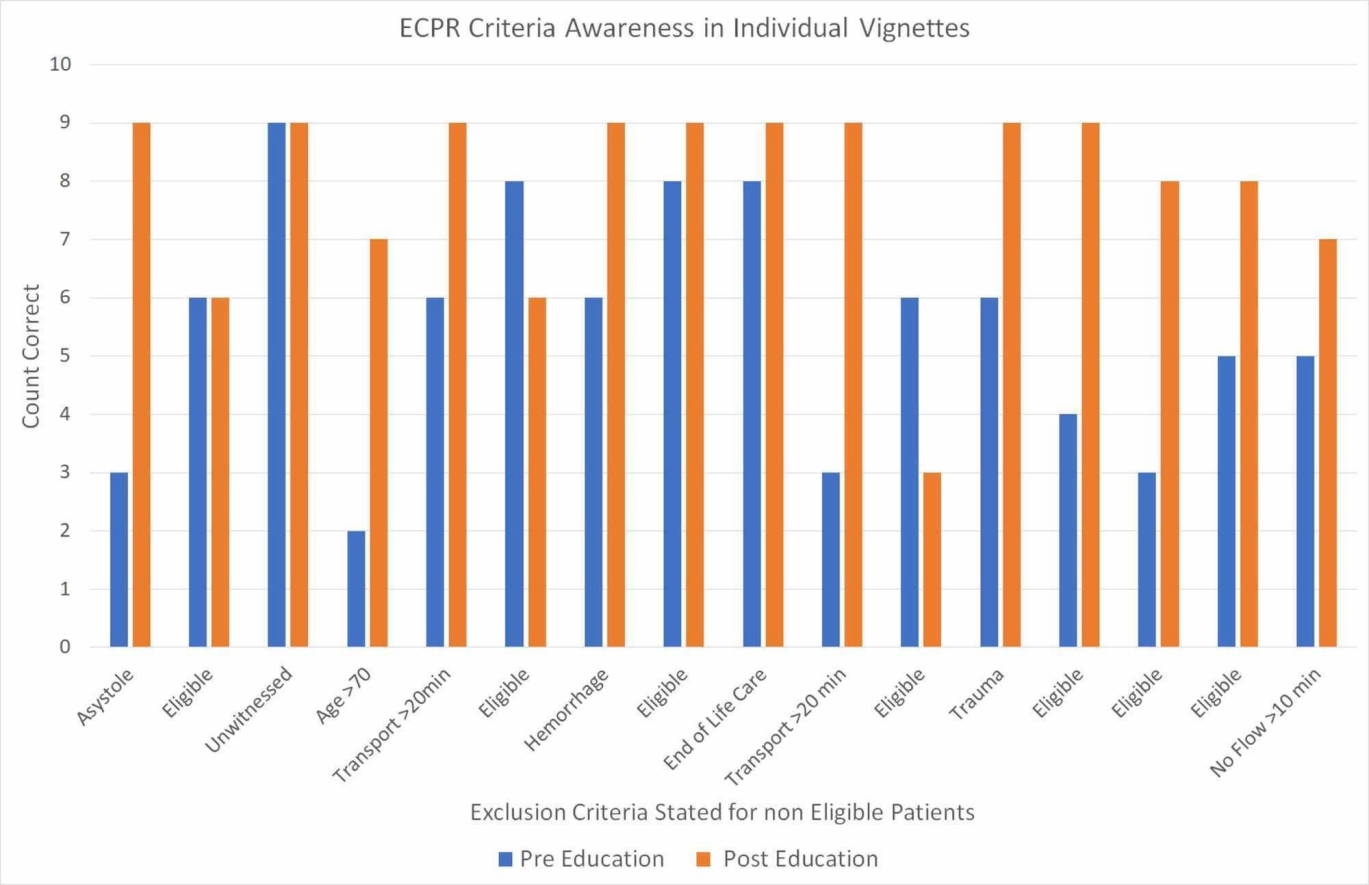
Aggregate summary of paramedic performance on individual survey questions. ECPR: Extracorporeal cardiopulmonary resuscitation

Aggregate paramedic responses to individual questions are demonstrated in Figure 2. After the education delivery, paramedic performance on individual scenarios improved or was neutral on 14 out of 16 scenarios (87.5%). In two scenarios where the patients were eligible for ECPR correct triage decreased after the education delivery. In the scenario with an unwitnessed cardiac arrest all paramedics correctly excluded the patient both before and after the education delivery. In the remaining eight scenarios with clear exclusion criteria, there was a higher proportion of correctly excluded patients after the education delivery.

Table 3 utilizes a contingency table to represent the triage decisions made by the paramedics in each patient scenario.

**Table 3:** Performance of paramedic ECPR activation for individual patient vignettes. Overtriage = a/(a + b) Undertriage = d/(b + d) ECPR: Extracorporeal cardiopulmonary resuscitation

	ECPR Non-Eligible Patient		ECPR Eligible Patient	Total
Protocol Activated	4 (a)		49 (b)	53
No Protocol Activation	77 (c)		14 (d)	91
Total	81		63	144
	Triage Performance			
	Overtriage	Undertriage		
	7.5%	22%		

Qualitative data collected through feedback from the paramedics during the simulation sessions included perceived potential difficulty using the mechanical chest compression system (MCCS) in extractions with narrow passageways which are a common feature of our local architectures. The paramedics also noted the need to develop strategies to troubleshoot MCCS device errors during transport. Refining the inclusion and exclusion criteria was also suggested to give the paramedics clear direction for the inclusion or exclusion of pregnant patients and pediatric patients.

## Discussion

The goal of this pilot study was to assess an EMS knowledge translation component required in the development of an ECPR protocol that will be implemented into our hospital and prehospital care systems. Specifically, we investigated whether an educational package consisting of a didactic lecture coupled with high fidelity simulation could improve paramedics’ ability to correctly triage patients who meet eligibility for implementation of ECPR. We found that prior to formal training paramedics were able to correctly triage appropriate ECPR patients over 60% of the time. This suggests that paramedics have a significant gestalt for identifying patients who are candidates for ECPR. Their performance improved to a correct triage rate of almost 90% after the delivery of our education package. Further testing is planned to assess paramedic retention in six months.

Review of the paramedics’ survey performance in the individual scenarios revealed a trend of improved triage in cases where clear exclusion criteria were present. Interestingly, in two scenarios where patients met the inclusion criteria, the paramedics’ performance showed a negative trend after the education delivery. In these scenarios, there was some ambiguous data in the vignette i.e.: it was unclear if adequate resuscitation (three cycles or 10 minutes of CPR) had been performed. Despite the ambiguity, there were no exclusion criteria present to make the patient ineligible for ECPR. This suggests that perhaps paramedics should only consider the exclusion criteria. This could increase the specificity of patient identification and avoid undertriage of patients who may be eligible. This would work well in the flow of our proposed protocol. An additional review of the inclusion and exclusion criteria would be performed by the attending physician at the receiving hospital before the patient would be placed on ECMO. Although there is no current consensus on acceptable overtriage and undertriage for ECPR, similar principles apply to the rational used for the accepted targets in trauma. The focus has been to minimize undertriage and the potential for associated morbidity and mortality. A goal of less than 5% undertriage and a range of 25-35% overtriage are the accepted standards in trauma [13]. The implementation of a simplified triage criteria for paramedics focusing on absolute contraindications should bring our undertriage and overtriage rates into better alignment with the published standards in trauma, although further testing will be required to confirm this hypothesis.

Simulation with paramedics allowed us to provide real time feedback during the simulated OHCA cases. In the simulations, the paramedic playing the role of ACP acted as the team leader. They coordinated the resuscitation and had the ECPR pocket card in hand during resuscitation. Closed loop communication was used between paramedics and a review of the eligibility criteria took place prior to the transport decision point. No measurable decrease in CPR performance was noted during the eligibility criteria check. The patient was attached to the MCCS device prior to the completion of three cycles of CPR or a total CPR time of 10 minutes. With the aid of the pocket card, paramedics correctly triaged patients in all simulated cases.

In the literature to date, it has been demonstrated that studies with a shorter time to initiation of ECMO have a higher percentage of patients with favorable outcomes [14]. Furthermore, implementation of an ECPR simulation program has been shown to reduce times to ECMO in real patients [15]. For these reasons it was essential to involve paramedics in the development of our local ECPR program. Having them simulate scenarios allowed us to better understand potential barriers for implementing the ECPR protocol. ECMO is a high acuity but low opportunity scenario. Building a malleable protocol that can evolve over time, involves appropriate stakeholders, and responds to any failures will be key to success [16]. We have implemented this strategy and continue to improve our protocol as we embark on our journey to bring this worthwhile service to the local population.

Limitations

The results of this pilot project must be interpreted with caution. It is a pilot project; it will contribute to the framework in developing a more robust training experience to providers participating in our ECPR program. It should not be interpreted in isolation but will provide context for the future research within our ECPR program. The numbers in this study are low and a formal power analysis was not performed, instead a convenience sampling was chosen. We attempted to involve as many local ACPs as possible and were successful in doing this, however, we were limited by the number of a local ACPs and a finite time period to complete this pilot project.

## Conclusions

We sought to determine if an educational program can improve identification of ECPR candidates by paramedics. Paramedic training through a didactic session coupled with a pocket card and simulation appeared to be a feasible method of knowledge translation. Establishing paramedic competence will ensure rapid transfer of eligible patients for ECMO, a potentially life-saving intervention. Six-month follow-up data will help ensure knowledge retention is achieved.

## Appendices

ECPR (Extracorporeal cardiopulmonary resuscitation) research project survey

This survey is being conducted as part of a research project within the Saint John Regional Hospital Department of Emergency Medicine Research Team. We are interested in determining if an educational tool can be used by paramedics to triage patients who may be eligible for treatment with ECPR (Extracorporeal cardiopulmonary resuscitation). The study has been approved by the Horizon Health Ethics Board (HHN REB File #: 2017-2487). To protect your privacy and ensure confidentiality, you are asked to create a unique identifier. Your unique identifier is created using: Mother’s initials using maiden name Last two numbers of personal telephone number Street number of home address Two-digit month of birth That is: Mother’s maiden name Marie Smith, 666-4333, 150 Right Street, March would give the unique identifier of (MS3315003). This unique identifier is collected to prevent accidental duplication of data and to facilitate comparison with the Exit Survey. No personally identifiable data will be collected, shared with third parties or published in any form. If you agree to participate in this research please select yes and you will be directed to the survey. If you do not wish to participate select no and you will be directed away from this page.

Please enter your unique identifier below *

Do you agree to participate in this study? *

Mark only one oval.

Yes

No

Demographics

Please select your gender *

Mark only one oval.

Female

Male

Other:

What is your current highest level of completed training? *

Mark only one oval.

Primary Care Paramedicine

Advanced Care Paramedicine

Critical Care Paramedicine

What is your age? *

Mark only one oval.

<25

25-34

35-44

45-54

55+

Scenario 1

1. A 55-year-old male with chest pain playing golf collapsed in the parking lot of Rockwood park golf course (<1 km away from SJRH). One of his golf partners is a nurse and could not palpate a pulse. He began immediate CPR. You arrive on scene 10 minutes after the cardiac arrest and take over the resuscitation. Asystole is identified on the cardiac monitor, you perform 3 cycles of CPR with no Return of Spontaneous Circulation (ROSC) and persistent asystole.

Would this patient be eligible for ECPR? *

Mark only one oval.

Yes

No

What is your next step? *

Mark only one oval.

Continue field resuscitation

Initiation of transport to the Saint John Regional Hospital (SJRH)

Scenario 2

2. A 45-year-old woman was shopping at the McAllister Mall (≈15 minute drive to the SJRH) when a cashier saw her fall to the ground. The cashier approached her, she was unresponsive so the cashier called for help. A bystander trained in CPR began chest compressions 6 minutes after she collapsed. A PCP team arrives 4 minutes after CPR initiation. VFib is identified on the monitor. CPR, Defibrillation, and SGA insertion are performed by the PCP team. You (ACP) arrive and continue resuscitation. Since your arrival you have identified persistent VFib on the cardiac monitor and have given a dose of epinephrine.

Is this patient a candidate for ECPR? *

Mark only one oval.

Yes

No

Would you continue to resuscitate this patient in the field or would you initiate transport to the Saint John Regional Hospital *

Mark only one oval.

Continued resuscitation in the field

Initiate transport to SJRH

Scenario 3

3. You are called to the scene of a motor vehicle accident on a back road in Saint John that is approximately a 20-minute drive from the SJRH. It's 2:00 am and another driver called 911 after coming upon an unwitnessed accident with a vehicle that had crashed into a guard rail. The second motorist found the person unresponsive in their car and began CPR. You arrive on scene and note asystole on the monitor.

Is this patient a candidate for ECPR? *

Mark only one oval.

Yes

No

What is your next step? *

Mark only one oval.

Provide care according to ANB General Trauma Management Guideline for cardiac arrest

Initiation of transport to the SJRH

Scenario 4

4. A 75-year-old man collapsed at a fast food restaurant located approximately 5 minutes from the SJRH by ambulance. There was no choking before he collapsed. CPR was started immediately by a bystander. You arrive on scene 5 minutes later and take over resuscitation. Pulseless Ventricular Tachycardia (pVT) is identified on the monitor. One round of CPR and defibrillation was performed on scene.

Is this patient a candidate for ECPR? *

Mark only one oval.

Yes

No

What are your next steps? *

Mark only one oval.

Continued resuscitation in the field

Initiation of transport to the SJRH

Scenario 5

5. A 51-year-old female collapses while at the Kingston Farmer’s Market (A 50-minute drive to the SJRH). A bystander who witnessed the collapse began immediate CPR. The nearest ambulance is already tending to another cardiac arrest, so your ambulance is dispatched. You arrive on scene 15 minutes after the patient collapses and take over resuscitation. After 10 minutes of resuscitation including defibrillation, airway insertion and administration of epinephrine the rhythm on the monitor shows VFib.

Is this patient a candidate for ECPR? *

Mark only one oval.

Yes

No

What is your next step? *

Mark only one oval.

Continue resuscitation in the field

Initiate transport to the SJRH

Scenario 6

6. A 62-year-old male collapses in King Square (a 10-minute ambulance ride to the SJRH). Bystander CPR is initiated within 8 minutes of the collapse. You arrive on scene 10 minutes after the collapse and take over resuscitation. Pulseless ventricular tachycardia is the first rhythm identified on the monitor followed by ventricular fibrillation at the next rhythm check. You have placed an advanced airway and given a dose of epinephrine while completing the first 2 cycles of CPR. Since you took over resuscitation 2 cycles of CPR/defibrillation have been completed and approximately 5 minutes have passed.

Is this patient a candidate for ECPR? *

Mark only one oval.

Yes

No

What would be your next step? *

Mark only one oval.

Continue with resuscitation in the field

Initiate transport to the SJRH

Scenario 7

7. You are dispatched to a home on the city’s west side (14-minute drive to the SJRH) with the report of a stabbing. When you arrive, a 35-year-old male patient is identified. He has a GCS of 7 and is suffering uncontrolled bleeding from multiple stab wounds. After initial assessment and administration of a fluid bolus the patient becomes unresponsive. PEA is observed on the monitor. You begin CPR.

Is this patient a candidate for ECPR? *

Mark only one oval.

Yes

No

What would be your next step? *

Mark only one oval.

Continue field resuscitation

Initiation of transport to the SJRH

Scenario 8

8. A 58-year-old woman collapsed at home in Milledgeville (7-minute transfer time to the SJRH) after clutching at her chest. A family member immediately began CPR. You arrive on scene within 10 minutes of the collapse. Pulseless Electrical Activity (PEA) is the initial rhythm on the monitor. You take over resuscitation and complete 3 cycles of CPR, place an advanced airway and have given 2 doses of epinephrine. At the next rhythm check Vfib is identified and you administer a shock without return of spontaneous circulation.

Is this patient a candidate for ECPR? *

Mark only one oval.

Yes

No

What is your next step? *

Mark only one oval.

Continue field resuscitation

Initiate transfer to the SJRH

Scenario 9

9. You are called to the home of a 64-year-old male on the city's east side (15-minute transfer to the SJRH) with a report that he has stopped breathing. When you arrive, his family tells you that the patient has ALS and has been receiving end of life care for the last month. You examine the patient and cannot feel a pulse VFib is the initial rhythm on the monitor.

Is this patient a candidate for ECPR? *

Mark only one oval.

Yes

No

What is your next step? *

Mark only one oval.

Confirm DNR status with family

Continue field resuscitation

Initiate transport to the SJRH

Scenario 10

10. A 47-year-old male collapses during a Men’s league basketball game in the Kennebecasis Valley (≈25-minute transfer to the SJRH). A teammate immediately begins CPR. You arrive on scene 10 minutes after the patient collapsed and pulseless VTach is the rhythm on the monitor. Ten minutes pass as you resuscitate the patient including CPR, defibrillation, airway placement and administration of 2 doses of epinephrine and 1 dose of amiodarone.

Is this patient a candidate for ECPR? *

Mark only one oval.

Yes

No

What is your next step? *

Mark only one oval.

Continued resuscitation in the field

Initiation of transport to the SJRH

Scenario 11

11. A 65-year-old male loses consciousness while working in a confined space (10 minutes from the hospital). The safety consultant monitoring him witnessed his collapse. It takes 6 minutes to extract the patient from the confined space. You have already arrived on scene and begin CPR immediately after the extraction. Asystole is the initial rhythm on the monitor but after 12 minutes of resuscitation (including multiple rounds of CPR, placement of a definitive airway, and 3 doses of Epinephrine) the monitor showed VFib on the last 2 rhythm checks and defibrillations were delivered without ROSC.

Is this patient a candidate for ECPR? *

Mark only one oval.

Yes

No

What is your next step? *

Mark only one oval.

Continued resuscitation in the field

Initiation of transport to the SJRH

Scenario 12

12. A 32-year-old woman was in a high speed MVC on the MacKay Highway (a 10-minute transfer to the SJRH). When you arrive on scene you find her ejected from the vehicle and comatose. Initial vitals are BP: 70/54 HR: 52 and RR:24 with shallow respirations. While attaching her to the spine board she stops breathing and you do not feel a pulse.

Is this patient a candidate for ECPR? *

Mark only one oval.

Yes

No

What is your next step? *

Mark only one oval.

Initiation of transport to the SJRH

Provide care according to ANB General Trauma Management Guideline for cardiac arrest.

Scenario 13

13. You are dispatched to the home of a patient (15 minutes transport time to the SJRH) who called 911 after having rapid onset of shortness of breath and chest pain. When you arrive, the family tells you that the patient (a 52-year-old male) collapsed 5 minutes ago and a family member has been performing CPR. You take over resuscitation. PEA is identified on the monitor. As you perform your resuscitation the family tells you he had a knee replacement 10 days ago and his right leg has been swollen and red. You complete 3 cycles of CPR with defibrillation, place an airway, and administer Epinephrine as indicated.

Is this patient a candidate for ECPR? *

Mark only one oval.

Yes

No

What is your next step? *

Mark only one oval.

Continue field resuscitation

Initiation of transport to the SJRH

Scenario 14

14. You are dispatched to the Renforth Warf (15-minute transport time to the SJRH). When you arrive, you find a 27-year-old female who fell through the ice and is partially submerged next to an ice fishing shack. She is non-responsive but appears to be taking very shallow respirations. Her fishing partner tells you that she stopped responding to him approximated 5 minutes ago. You get her to land and hook her up to the monitor, the initial rhythm identified on the monitor is PEA. You resuscitate the patient for 10 minutes.

Is this patient a candidate for ECPR? *

Mark only one oval.

Yes

No

What is your next step? *

Mark only one oval.

Continued field resuscitation according to ANBs Hypothermia/Cold Exposure guideline

Initiation of transport to the SJRH

Scenario 15

15. You are called to the home of a 33-year-old man who overdosed on a β-blocker and antipsychotic medication. When you arrive, you find him somnolent and taking shallow respirations. When you attach the cardiac monitor severe bradycardia is identified and after a minute the rhythm degrades into VFib. You spend the next 6 minutes performing 2 cycles of CPR including airway placement, Epinephrine administration and defibrillation. The transport time to hospital is estimated to be 15 minutes.

Is this patient a candidate for ECPR? *

Mark only one oval.

Yes

No

What is your next step? *

Mark only one oval.

Continued resuscitation in the field

Initiation of transport to the SJRH

Scenario 16

16. A 52-year-old woman collapses while babysitting her 5 year old grandchild. The grandchild calls 911 immediately and you are dispatched. You arrive on scene 13 minutes from the time of collapse and begin CPR. Vfib is the initial rhythm identified on the monitor you perform CPR for 11 minutes with airway placement, defibrillation, and administration of Epinephrine and Amiodarone. The estimated transport time to hospital is 20 minutes.

Is this patient a candidate for ECPR? *

Mark only one oval.

Yes

No. After the last question in this section, stop filling in this form.

What is your next step? *

Mark only one oval.

Continued field resuscitation

Initiation of transport to the SJRH
